# Epstein-Barr Virus and Multiple Sclerosis

**DOI:** 10.3389/fimmu.2020.587078

**Published:** 2020-12-17

**Authors:** Gunnar Houen, Nicole Hartwig Trier, Jette Lautrup Frederiksen

**Affiliations:** ^1^ Institute of Biochemistry and Molecular Biology, University of Southern Denmark, Odense, Denmark; ^2^ Department of Neurology, Rigshospitalet, Glostrup, Denmark; ^3^ Institute of Clinical Medicine, University of Copenhagen, Copenhagen, Denmark

**Keywords:** Epstein-Barr virus, multiple sclerosis, immune evasion, central nervous system, chronic infection, relapsing-remitting

## Abstract

Multiple sclerosis (MS) is a neurologic disease affecting myelinated nerves in the central nervous system (CNS). The disease often debuts as a clinically isolated syndrome, e.g., optic neuritis (ON), which later develops into relapsing-remitting (RR) MS, with temporal attacks or primary progressive (PP) MS. Characteristic features of MS are inflammatory foci in the CNS and intrathecal synthesis of immunoglobulins (Igs), measured as an IgG index, oligoclonal bands (OCBs), or specific antibody indexes. Major predisposing factors for MS are certain tissue types (e.g., HLA DRB1*15:01), vitamin D deficiency, smoking, obesity, and infection with Epstein-Barr virus (EBV). Many of the clinical signs of MS described above can be explained by chronic/recurrent EBV infection and current models of EBV involvement suggest that RRMS may be caused by repeated entry of EBV-transformed B cells to the CNS in connection with attacks, while PPMS may be caused by more chronic activity of EBV-transformed B cells in the CNS. In line with the model of EBV’s role in MS, new treatments based on monoclonal antibodies (MAbs) targeting B cells have shown good efficacy in clinical trials both for RRMS and PPMS, while MAbs inhibiting B cell mobilization and entry to the CNS have shown efficacy in RRMS. Thus, these agents, which are now first line therapy in many patients, may be hypothesized to function by counteracting a chronic EBV infection.

## Introduction

Multiple sclerosis (MS) is a disease affecting the central nervous system (CNS), with inflammation and demyelination of nerves, eventually resulting in nerve damage and disabilities. MS can take different courses, most often in the form of relapsing-remitting (RR) cycles of disease activity or more rarely as a primary-progressive (PP) disease. RR MS can progress over many years and may eventually develop into a secondary-progressive (SP) disease (1–3).

Initial symptoms of MS are often recorded as solitary symptoms, i.e., a clinically isolated syndrome in the form of optic neuritis (ON) or other neurological disturbances isolated in time and space (1–4). Diagnosis of MS relies on the so-called McDonald criteria, latest updated in 2017 (5). These criteria include detection of active inflammatory foci in the CNS as seen by positron emission tomography (PET) and magnetic resonance imaging (MRI) and intrathecal production of immunoglobulins (Igs), measured as an elevated cerebrospinal fluid (CSF)/serum IgG index, as a free light chain index or as the occurrence of oligoclonal bands (OCBs) of IgG in CSF (6–10). Each oligoclonal band is a result of intrathecal antibody (Ab) synthesis by single B cell clones and therefore, specific CSF/serum Ab indices (AIs) may also be elevated, e.g., Abs to various viruses, corresponding to the specificity of some of the OCBs (11–15). Accordingly, the OCB Abs show evidence of antigen (Ag) exposure, somatic hypermutation and affinity maturation (16–19).

Differential diagnoses for MS are neuromyelitis optica (NMO) and major oligodendrocyte glycoprotein (MOG) Ab-associated demyelinating disease, but other diseases may also mimic some aspects of MS, including acute disseminated encephalomyelopathy (ADEM), CNS neoplasms and various other diseases with the potential to affect the CNS (20–22).

Therapy of MS was previously mainly empirical and relied on several low molecular weight (LMW) drugs, including glatiramer acetate, teriflunomide, dimethyl fumarate, fingolimod, cladribine and others, however, biological drugs have been introduced for treatment of RRMS, including beta-interferon and several therapeutic monoclonal Abs (MAbs) (23–25). Especially the array of MAbs approved for MS treatment has expanded and currently range from Natalizumab, an integrin α4β1/α4β7 MAb, Alemtuzumab, a CD52 Mab, to MAbs targeting the B cell surface marker CD20 (Rituximab, Ocrelizumab) (25–28). Most interestingly, the latter have been found to have an effect also on PPMS (27, 28).

## MS Etiology and Epidemiology

No consensus about MS etiology exists at present and theories range from idiopathic loss of self-tolerance, over molecular mimicry to chronic virus infections. However, it is generally accepted that MS involves a combination of genetic predisposing factors and environmental influences (29–34). MS has a female preponderance, which most likely is due to genetic factors and incidence is highest after puberty, which may be ascribed to either genetic or environmental factors or both.

Genetic factors influencing development of MS are in particular major histocompatibility class II (MHC II) alleles, of which some increase susceptibility (e.g., human leukocyte antigen (HLA) DRB1*15:01), while others decrease susceptibility. Likewise, some MHC I alleles also appear to be protective (e.g., HLA A*02.01), while others increase susceptibility. Overall, more than 100 genes have been found to have an influence on development of MS, of which most are involved in immune system functioning and in particular lymphocyte and Ab functioning (1–3, 29–40).

Environmental factors with an impact on MS incidence include sunlight exposure/vitamin D (vitD) deficiency, dietary and other compounds, smoking and some virus infections [e.g., Epstein-Barr Virus (EBV)] (30).

MS is most prevalent on the Northern hemisphere, a finding which can most likely be related to the intensity of sun light, which may in turn be explained by levels of vitD synthesis. Actually, vitD concentrations have been found to be correlated with MS incidence/prevalence (39, 41–43).

Smoking increases the risk of MS, but some other uses of tobacco may actually reduce the risk of MS (30, 44–46). Other environmental compound exposures have been found to have an effect om MS susceptibility (30) and recently, propionic acid and the composition of the intestinal microbiota has been reported to influence or be influenced by MS (47–49).

Obesity, especially in adolescence has been reported to have an effect on MS susceptibility, but it is unclear whether this may be attributed to genetically determined factors or environmental/socio-economical influences or a combination of different effects, e.g., a low-grade neuro-inflammatory effect or a vitD-sequestering effect (50–53).

Virus infections have for long been suspected to be involved in MS development (29–32, 54–56). Most investigations have focused on EBV, which remains the most likely candidate for a causative virus, but other viruses may also play a role as discussed below.

## Epstein-Barr Virus (EBV)

EBV is a member of the Human Herpes Virus (HHV) family, which also includes Herpes Simplex Virus (HSV) 1 and 2, Varicella Zoster Virus (VZV), Cytomegalovirus (CMV), HHV 6 and 7, and Kaposi Sarcoma Virus (KSV) (57–59). EBV is an enveloped virus with a 120 kB double-stranded DNA genome, coding for about 85 proteins and a number of non-coding RNAs (60–65).

EBV is transmitted to new victims with saliva and infects pharyngeal epithelial cells. When released from the epithelial cells, EBV infects B cells in the associated underlying tissue, where it may be propagated or enter a state of latency, depending on the B cell environment and the state of the host immune response (66–70). Initially, in the absence of an adaptive immune response, B cells are induced to lytic production of virus. Upon entry to the cell, EBV uncoats in the cytoplasm and transfers its DNA to the nucleus, where an ordered sequence of viral gene expression then takes place. First, immediate early genes are expressed, coding for transcription factors and other proteins involved in control of the host cell, next early genes are expressed, coding for proteins involved in viral DNA replication, followed by late genes, coding for capsid proteins and other proteins involved in mature virus production [e.g., envelope (glyco)proteins)]. Finally, virions are released from the cell by a process resembling the reverse of endocytosis. At later stages, when an adaptive immune response has been established, EBV may enter a latent state, where only few or no viral genes are expressed, but the viral genome may still be replicated along with cellular DNA. This state is called “deep” latency, where from the virus may be reactivated in response to B cell activation (66, 71–80).

As a counter-measure to host immune responses, EBV has evolved a multitude of immune evasion mechanisms, counteracting both host cell intracellular anti-viral processes and host extracellular innate and adaptive immune responses. Cellular anti-viral pathways are many and EBV devotes a large part of its genome to control of cellular anti-viral apoptosis mechanisms and to immune evasion (81–86).

The adaptive immune response to EBV involves both Ab-dependent processes and cytotoxic T cells, and EBV has evolved mechanisms to evade these as described above, e.g., by down-regulating MHC I to avoid recognition by cytotoxic T cells. Therefore, control of EBV relies to a large extent on natural killer cell surveillance of infected cells with too little MHC I on the surface, which is in turn counter-balanced by EBV by upregulation of non-classical MHC molecules (87–102).

Despite the many evasion mechanisms of EBV, the host immune system eventually forces EBV into latency, where a minimal number of EBV genes are expressed as described above. However, T cell immunity eventually wanes with time, allowing EBV to reactivate under certain conditions with lytic production of virions, thus re-invigorating the immune response, again forcing the virus into latency, a cyclic process which may go on for the rest of a person’s life with smaller or larger intervals, depending on the person’s immune system profile.

Decreased capacity for immune control of EBV may, in some cases manifest itself as a tendency to develop EBV-related diseases, including infectious mononucleosis (IM), various cancers, MS, and other relapsing-remitting autoimmune diseases (e.g., systemic autoimmune diseases) (103–112).

## EBV and MS

In MS, much evidence indicates a role for EBV and specifically that EBV-infected B cells have entered the CNS at some point of disease development (**Table 1**). As described above, some of the major characteristics of MS are the presence of an elevated IgG index and OCBs in the CNS, representing various B cell clones synthesizing Abs in the CNS (6–8). The elevated IgG index and the OCBs cannot reflect simple diffusion of Abs from serum to CSF, since the IgG index is calculated relative to the albumin ratio and the OCBs test is only regarded as positive, when the OCBs are absent from serum. Similarly, intrathecal presence of elevated free light chains represent synthesis of Abs in the CNS (9, 10). Intrathecal synthesis of Abs is also reflected in elevated specific antibody indexes (AIs), representing intrathecal synthesis of Abs to Measles Virus (MeV) antigens (Ags), Mumps Virus (MuV) Ags, HZV Ags, Rubella Virus (RuV) Ags, and other pathogen Ags (11–16). EBV AIs are also elevated, however, not necessarily to the same extent as other AIs, despite the presence of high levels of Abs to EBV in serum of MS patients (15, 124). Interestingly, there is a high degree of correlation between Ab concentrations in serum and in CSF for most or all of the virus Abs described above (15). Since the elevated CSF levels are not caused by diffusion from serum to CSF and since there is a highly significant correlation between serum and CSF Ab levels, the only likely explanation is that there has been or is a continuous influx of Ab-producing B cells from blood to CSF, most likely in the form of B cell blasts which have differentiated to plasma cells concomitantly in the periphery and in the CNS.

**Table 1:** Evidence for Epstein-Barr virus (EBV) involvement in multiple sclerosis (MS).

MS trait/characteristic	EBV relation	References
Elevated IgG index	CNS entry of EBV-infected B cells and differentiation to plasma cells	(6)
OCBs in CSF	CNS entry of EBV-infected B cells and differentiation to plasma cells	(6, 8)
Elevated FLCs	CNS entry of EBV-infected B cells and differentiation to plasma cells	(9, 10)
Elevated specific AIs	CNS entry of EBV-infected B cells and differentiation to plasma cells	(11–19)
CNS inflammatory foci	T cell attack on CNS EBV-infected B cells	(1, 2, 5)
Demyelination in CNS	Inflammatory damage to oligodendrocytes and stimulation of macrophages and microglia cells	(1–3)
AuAbs to myelin AuAgs	Inflammation-induced stimulation of (EBV-infected) B cells and damage to oligodendrocytes	(113–116)
Therapy with CD20 MAbs	Killing of EBV-infected B cells, prevention of CNS entry	(27, 28)
Therapy with integrin MAbs	Prevention of CNS entry of EBV-infected B cells	(117, 118)
Therapy with EBV-specific T cells	Killing of EBV-infected B cells, prevention of CNS entry	(119, 120)
Female preponderance	Reduced EBV control (immune suppression due to menstruation (blood loss, healing, hormonal factors)	(1–3, 30)
Incidence increases after puberty	Increased exposure to EBV, reduced capacity for EBV control due to thymus involution	(3)
HLA DRB1 predisposes	Increased entry and/or decreased immune control of EBV	(1–3, 29–40)
IM predisposes	Increased load of EBV-transformed B cells	(30, 54–56, 121–123)
VitD deficiency predisposes	Reduced EBV control (immune suppression due to vitD deficiency of leukocytes, (e.g., T cells, NK cells)	(39, 41–43)
Smoking predisposes	Reduced EBV control (immune suppression by smoke) and/or increased frequency of EBV reactivation	(30, 44–46)
Obesity predisposes	Reduced EBV control due to immune suppression	(50–53)

Ab, Antibody; Ag, antigen; AI, antibody index; AuAb, autoantibody; AuAg, autoantigen; CD, cluster of differentiation; CNS, central nervous system; EBV, Epstein-Barr virus; FLC, free light chains; HLA, human leukocyte antigen; IM, infectious mononucleosis; MAb, monoclonal antibody; NK, natural killer; VitD, vitamin D.

Many studies have revealed increased amounts and increased frequencies of EBV Abs in MS, however, such studies are hampered by the nearly ubiquitous presence of EBV in adults. Moreover, the results seem to depend somewhat on the EBV Ags used and the assay methodology.

Seroconversion from negative to positive for EBV Abs generally increases with age. It has a major incidence peak early in childhood and shows a second peak, especially for females, around puberty, co-incident with the approximate age of IM and co-incident with the female predominance in MS (3, 103, 104, 106, 125–128). EBV infection correlates with pediatric MS and essentially all children with MS are found to be positive for EBV Abs, whereas the positivity rate is considerably lower in healthy children (54, 129–132). When using an array of Ags and methods, all adult MS patients are also found to be positive for EBV Abs and it appears that MS development generally depends on prior EBV infection (54–56, 121, 122, 130, 133–137). Furthermore, prior IM has been found to increase the risk of MS by more than 2-fold by itself and more in combination with other predisposing parameters (30, 54–56, 121–123, 138, 139).

In contrast to the Ab-based studies, polymerase chain reaction (PCR)-based investigations on EBV DNA and RNA in blood, CSF and saliva have generally shown no or only minor differences between MS patients and controls (140–142). These results may depend on the patient cohorts and the methods employed, but they do indicate that the role of EBV in MS reflects a predominantly latent infection (as in most infected persons) with occasional reactivation and transient lytic virus production. However, sequencing-based studies have indicated an association between the presence of EBV variants and MS (143, 144).

*In situ* hybridization and PCR studies on brain material from MS patients have in some cases indicated the presence of EBV DNA in lesions, but other studies have yielded negative results (145–148). Immuno-histochemical studies are few, but one study has demonstrated the presence of EBV Ags in post-mortem brain tissue of MS patients (149).

Other viruses, including RuV, MuV, MeV, CMV, HHV6, VZV, John Cunningham Virus (JCV), and Human Endogenous Retrovirus W (HERV-W) have also been suggested to play a role in MS, either by themselves or in combination with EBV infection (30, 54, 150–154). This may simply reflect a viral Ag-induced reactivation and stimulation of EBV-infected B cells with specificity for the virus(es) in question (i.e., a secondary role for these viruses), or it may reflect a more active role of the viruses. The virus Ab profile varies much between individual patients, thus favoring a primary role of EBV and a secondary role of other viruses (15). Interestingly, CMV seropositivity appears to afford some protection against MS development (30, 135). CMV is evolutionarily related to EBV, so it may be a likely possibility that CMV may exhibit some cross-reactivity with and protection against EBV (59).

As described above, EBV control relies to a large extent on T cells and NK cells. It could therefore be hypothesized that MS patients have a deficiency in the cellular immune control of EBV and possibly also other viruses. CD8 T cell infiltration of MS brain lesions has been demonstrated in several studies but defective T cell control of EBV has also been reported in MS patients (155–157). This could indicate an imbalance in the T cell control of EBV in MS patients, and one study has actually found increased programmed death (PD) 1 on CD8 T cells with resulting decreased cytolytic activity against EBV-infected B cells (158), while PD1 has also been reported to be increased on regulatory T cells (159).

## Discussion

MS has traditionally been regarded as an autoimmune disease. However, the occurrence of autoantibodies (AuAbs) in MS (e.g., myelin basic protein (MBP) and major oligodendrocyte glycoprotein (MOG) Abs) is limited to only some patients and the pathogenic role of AuAbs remains debatable, while the search for autoantigens (AuAgs) in MS continues (113–116, 160–173). For this reason, models of MS etiology have for long revolved around T cells as major contributors. The role of T cells has been suggested to involve idiopathic loss of self tolerance with expansion of self-reactive T cell clones, defective regulatory T cells, infections in combination with (T cell) molecular mimicry and epitope spreading, bystander T cell activation, exhaustion of infection-related T cells, or combinations/imbalances of these (1–3, 30, 54, 173–182). Even though EBV-infected B cells appear to play a major role in MS, is an important role for T cells not excluded. EBV-infected memory B cells will be sensitive to stimulation by both their cognate Ags and specific CD4-positive T helper cells and will be a target for CD8-positive cytotoxic T cells. Both stimulation by T helper cells and attack by cytotoxic T cells will contribute to inflammation around EBV-infected B cells. Thus, a major role for T cells in MS is likely, in agreement with the predominance of T cells in MS lesions (1, 2, 173–182).

**Figure 1:**
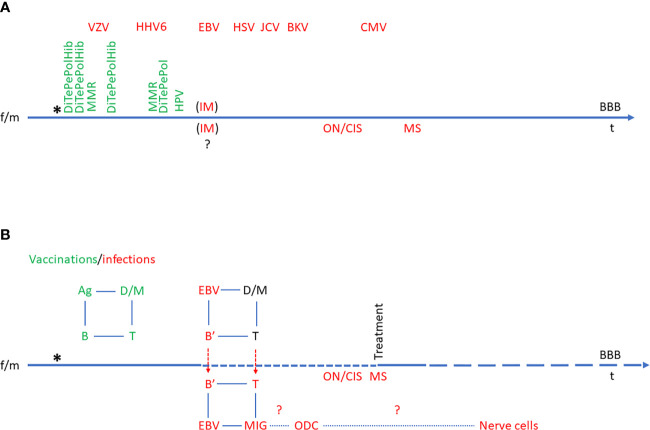
Model of Epstein-Barr virus (EBV)’s role in multiple sclerosis (MS). The time line also represents the blood-brain-barrier (BBB) and events across the BBB. The birth of a child subsequent to the mixing of a female (f) and a male (m) set of genes is indicated by an asterisk (*). **(A)** Time course of normal immune system development with vaccinations (green) and infections (red). The order and time course of vaccinations is defined by vaccination regimens. The order of infections is individual and variable, so the sequence indicated is hypothetical. In some individuals, EBV infection may manifest itself as IM, and it is not known to which extent infectious mononucleosis (IM) affects the CNS at the time of primary infection, but it is known to increase the risk of ON/CIS and eventually MS. **(B)** Schematic presentation of etiological immunological reactions in multiple sclerosis in relation to vaccinations and infections. The normal immunological feed-back loop is indicated in green (e.g., vaccination-induced Ag uptake by dendritic cells (D) and macrophages (M), which interact with T cells, which in turn interact with B cells and vice versa). In the case of EBV infection, the immunological feed-back loop is re-programmed to the advantage of EBV, resulting in chronic infection of B cells (B’). These may enter the CNS (particularly in the case of IM) and be followed by T cells. This results in inflammation in the CNS with the feed-back loop also involving microglia cells (MIG) and at some point also oligodendrocytes (ODC) and eventually, nerve cells. Ag, antigen; B, B cell; B’, EBV-infected B cell; BBB, blood-brain barrier; BKV, B. K. Virus infection; CIS, clinically isolated syndrome; CMV, Cytomegalovirus infection; D, dendritic cell; DiTePePolHib, Diphtheria-Tetanus-Pertussis-Polio-Hemophilus influenzae B vaccine; EBV, Epstein-Barr virus infection; f, female; HHV6, Human Herpes Virus 6 infection; HPV, Human Papilloma Virus vaccine; HSV, Herpes Simplex Virus infection; JCV, John Cunningham Virus infection; IM, infectious mononucleosis; m, male; M, macrophage; MIG, microglia cell; MMR, Measles-Mumps-Rubella vaccine; MS, multiple sclerosis; ODC, oligodendrocyte; ON, optic neuritis; t, time; T, T cell; VZV, Varicella Zoster Virus infection.

Thus, exhaustion of cytotoxic T cells and/or NK cells would seem to be highly relevant in relation to EBV involvement in MS as indicated above. This view has gained momentum from the relatively big success of B cell-targeted therapies in MS and CD20 MAbs are now the choice of treatment in many newly diagnosed MS patients (27, 28). These drugs can be hypothesized to work either by elimination of self-reactive B cell clones or elimination of EBV-infected (memory) B cells. As the frequencies of AuAbs in MS are variable and as CD20 is not expressed on differentiated Ab-producing “plasma” B cells, the first possibility can be regarded as more hypothetical (although a contribution of this to therapeutic outcome remains a possibility). Consequently, the second possibility, elimination of EBV-infected memory B cells, appears to be the most likely mechanism for the therapeutic effects of CD20 MAbs. The results described above indicate that EBV-transformed B cells proliferate or have proliferated in the periphery and entered the CNS at some point of disease evolution in connection with relapses (RRMS) or have entered the CNS at some point in disease evolution (SPMS and PPMS) (**Figure 1**). CD20-targeted MAbs are administered intravenously and are not expected to enter the CNS to any major degree (in line with the occurrence of CNS OCBs and elevated IgG index not deriving from diffusion from the blood stream). Therefore, the efficacy of these drugs must derive from an effect on CD20-positive B cells in the periphery, both in RRMS and PPMS, indicating that the import of EBV-transformed B cell to the CNS is a continuous process.

Other treatments with an effect in MS can also be related to a role of EBV. Natalizumab inhibits lymphocyte mobilization and entry to the CNS by targeting integrin α4β1/α4β7 (117, 183). Integrins may be used by EBV as entry receptors (118) and Natalizumab might therefore both inhibit entry of EBV to integrin-expressing cells and may also inhibit mobilization and entry of EBV-infected B cells and EBV-directed T cells to the CNS by a general inhibition of lymphocyte trafficking.

Some other low molecular weight MS drugs have also been reported to have an effect on EBV, in particular Teriflunomide, which has been reported to inhibit EBV lytic replication and to influence the immune response to EBV (118, 184). Similarly, the role of vitD in MS can be regarded as a general immune-stimulatory effect as can other environmental factors (e.g., propionic acid, which has been found to reactivate EBV (thus re-invigorating an EBV-targeted immune response) (119). Smoking can theoretically affect the disease course both by reducing immunity and by reactivating EBV, two effects that may partly oppose each other, thus possibly explaining the apparently protective role of some uses of tobacco (54).

In line with the role of EBV, small trials of MS therapy with autologous *in vitro*-expanded EBV-specific T cells have shown a beneficial effect in some patients (119, 185). The theory of EBV involvement in MS was proposed early by Pender et al. and it has been made likely that MS patients have a deficient T cell control of EBV-infected cells (54, 120, 155, 186–197). The theory of EBV involvement in MS has subsequently been elaborated and substantiated by many studies as described above and summarized in **Table 1**. Several models have been proposed based on the accumulated evidence for the role of EBV in MS (198–201). **Figure 1** represents an attempt to visualize much of this evidence.

In conclusion, the infectious, transforming, anti-apoptotic and immune-evasion properties of EBV makes it a highly likely candidate for an etiologic agent in MS. However, much remains to be investigated in future studies. For example, MS shows characteristics of an indolent neoplastic disease (metastasis, clonal expansion, overlap with lymphoma, etc.). Thus, the role of the transforming properties of EBV in MS should deserve attention. If the pathogenic role of EBV-specific T cell exhaustion can be confirmed, treatment of MS with immune check point inhibitors (e.g., PD1 and/or PD1 ligand (PD1L) MAbs), known to be effective in several forms of cancer may become a possibility.

## Author Contributions

GH made the first manuscript draft. All authors contributed to the article and approved the submitted version.

## Funding

The work was supported by grants from Stibofonden and Oda and Hans Svenningsens Fond.

## Conflict of Interest

The authors declare that the research was conducted in the absence of any commercial or financial relationships that could be construed as a potential conflict of interest.

## Abbreviations

Ab, antibody; ADEM, acute disseminated encephalomyelitis; Ag, antigen; AI, antibody index; AuAb, autoantibody; AuAg, autoantigen; B, B cell; B’, EBV-infected B cell; BBB, blood-brain barrier; BKV, B. K. Virus; CD, cluster of differentiation; CIS, clinically isolated syndrome; CMV, Cytomegalovirus; CNS, central nervous system; CSF, cerebrospinal fluid; D, dedritic cell; Di, Diphteria; EBV, Epstein-Barr Virus; f, female; FLC, free light chains; HERV, Human Endogenous Retrovirus; Hib, Hemophilus influenzae B; HHV, Human Herpes Virus; HLA, human leukocyte antigen; HPV, Human Papilloma Virus; HSV, Herpes Simplex Virus; Ig, immunoglobulin; IM, infectious mononucleosis; JCV, John Cunningham virus; KSV, Kaposi Sarcoma Virus; L, ligand; LMW, low molecular weight; M, macrophage; m, male; MAb, monoclonal antibody; MIG, microglia cell; MMR, Measles-Mumps-Rubella; MOG, major oligodendrocyte glycoprotein; MRI, magnetic resonance imaging; MS, multiple sclerosis; MuV, Mumps virus; NMO, neuromyelitis optica; OCB, oligoclonal bands; ODC, oligodendrocyte; ON, optic neuritis; PCR, polymerase chain reaction; PD, programmed death; Pe, pertussis; PET, positron emission tomography; Pol, polio; PP, primary-progressive; RR, relapsing-remitting; RuV, Rubella Virus; SP, secondary-progressive; T, T cell; t, time; Te, tetanus; VitD, vitamin D; VZV, Varicella Zoster Virus.

## References

[1] Dobson R Giovannoni G Multiple sclerosis - a review. Eur J Neurol (2019) 26:27–44. 10.1111/ene.13819 30300457

[2] Filippi M Bar-Or A Piehl F Preziosa P Solari A Vukusic S Multiple sclerosis. Nat Rev Dis Primers (2018) 4:43. 10.1038/s41572-018-0041-4 30410033

[3] Langille MM Rutatangwa A Francisco C Pediatric Multiple Sclerosis: A Review. Adv Pediatr (2019) 66:209–29. 10.1016/j.yapd.2019.03.003 31230695

[4] Kale N Optic neuritis as an early sign of multiple sclerosis. Eye Brain (2016) 8:195–202. 10.2147/EB.S54131 28539814 PMC5398757

[5] Thompson AJ Banwell BL Barkhof F Carroll WM Coetzee T Comi G Diagnosis of multiple sclerosis: 2017 revisions of the McDonald criteria. Lancet Neurol (2018) 17:162–73. 10.1016/S1474-4422(17)30470-2 29275977

[6] Simonsen CS Flemmen HØ Lauritzen T Berg-Hansen P Moen SM Celius EG The diagnostic value of IgG index versus oligoclonal bands in cerebrospinal fluid of patients with multiple sclerosis. Mult Scler J Exp Transl Clin (2020) 6:2055217319901291. 10.1177/2055217319901291 32030196 PMC6977237

[7] Arrambide G Tintore M Espejo C Auger C Castillo M Río J The value of oligoclonal bands in the multiple sclerosis diagnostic criteria. Brain (2018) 141:1075–84. 10.1093/brain/awy006 29462277

[8] Makhani N Lebrun C Siva A Narula S Wassmer E Brassat D Observatoire Francophone de la Sclérose en Plaques (OFSEP), Société Francophone de la Sclérose en Plaques (SFSEP), the Radiologically Isolated Syndrome Consortium (RISC) and the Pediatric Radiologically Isolated Syndrome Consortium (PARIS). Oligoclonal bands increase the specificity of MRI criteria to predict multiple sclerosis in children with radiologically isolated syndrome. Mult Scler J Exp Transl Clin (2019) 5:2055217319836664. 10.1177/2055217319836664 30915227 PMC6429663

[9] Gaetani L Di Carlo M Brachelente G Valletta F Eusebi P Mancini A Cerebrospinal fluid free light chains compared to oligoclonal bands as biomarkers in multiple sclerosis. J Neuroimmunol (2020) 339:577108. 10.1016/j.jneuroim.2019.577108 31743879

[10] Altinier S Puthenparampil M Zaninotto M Toffanin E Ruggero S Gallo P Free light chains in cerebrospinal fluid of multiple sclerosis patients negative for IgG oligoclonal bands. Clin Chim Acta (2019) 496:117–20. 10.1016/j.cca.2019.06.016 31233736

[11] Brettschneider J Tumani H Kiechle U Muche R Richards G Lehmensiek V IgG antibodies against measles, rubella, and varicella zoster virus predict conversion to multiple sclerosis in clinically isolated syndrome. PloS One (2009) 4:e7638. 10.1371/journal.pone.0007638 19890384 PMC2766627

[12] Jarius S Eichhorn P Franciotta D Petereit HF Akman-Demir G Wick M The MRZ reaction as a highly specific marker of multiple sclerosis: re-evaluation and structured review of the literature. J Neurol (2017) 264:453–66. 10.1007/s00415-016-8360-4 28005176

[13] Hottenrott T Dersch R Berger B Rauer S Huzly D Stich O The MRZ reaction in primary progressive multiple sclerosis. Fluids Barriers CNS (2017) 14:2. 10.1186/s12987-016-0049-7 28166789 PMC5294835

[14] Feki S Gargouri S Mejdoub S Dammak M Hachicha H Hadiji O The intrathecal polyspecific antiviral immune response (MRZ reaction): A potential cerebrospinal fluid marker for multiple sclerosis diagnosis. J Neuroimmunol (2018) 321:66–71. 10.1016/j.jneuroim.2018.05.015 29957390

[15] Houen G Heiden J Trier NH Draborg AH Benros ME Zinkevičiūte R Antibodies to Epstein-Barr Virus and neurotropic viruses in multiple sclerosis and optic neuritis. J Neuroimmunol (2020) 346:577314. 10.1016/j.jneuroim.2020.577314 32682138

[16] Reiber H Kruse-Sauter H Quentin CD Antibody patterns vary arbitrarily between cerebrospinal fluid and aqueous humor of the individual multiple sclerosis patient: specificity-independent pathological B cell function. J Neuroimmunol (2015) 278:247–54. 10.1016/j.jneuroim.2014.11.013 25468769

[17] Beltrán E Obermeier B Moser M Coret F Simó-Castelló M Boscá I Intrathecal somatic hypermutation of IgM in multiple sclerosis and neuroinflammation. Brain (2014) 137:2703–14. 10.1093/brain/awu205 25060097

[18] Obermeier B Mentele R Malotka J Kellermann J Kümpfel T Wekerle H Matching of oligoclonal immunoglobulin transcriptomes and proteomes of cerebrospinal fluid in multiple sclerosis. Nat Med (2008) 14:688–93. 10.1038/nm1714 18488038

[19] Qin Y Duquette P Zhang Y Olek M Da RR Richardson J Intrathecal B-cell clonal expansion, an early sign of humoral immunity, in the cerebrospinal fluid of patients with clinically isolated syndrome suggestive of multiple sclerosis. Lab Invest (2003) 83:1081–8. 10.1097/01.lab.0000077008.24259.0d 12861047

[20] Wingerchuk DM Weinshenker BG Neuromyelitis optica spectrum disorder diagnostic criteria: Sensitivity and specificity are both important. Mult Scler (2017) 23:182–4. 10.1177/1352458516688352 28080211

[21] Alves Do Rego C Collongues N Neuromyelitis optica spectrum disorders: Features of aquaporin-4, myelin oligodendrocyte glycoprotein and double-seronegative-mediated subtypes. Rev Neurol (Paris) (2018) 174:458–70. 10.1016/j.neurol.2018.02.084 29685427

[22] Miller DH Weinshenker BG Filippi M Banwell BL Cohen JA Freedman MS Differential diagnosis of suspected multiple sclerosis: a consensus approach. Mult Scler (2008) 14:1157–74. 10.1016/S1474-4422(17)30470-2 PMC2850590 18805839

[23] Tintore M Vidal-Jordana A Sastre-Garriga J Treatment of multiple sclerosis - success from bench to bedside. Nat Rev Neurol (2019) 15:53–8. 10.1038/s41582-018-0082-z 30315270

[24] Eriksson I Komen J Piehl F Malmström RE Wettermark B von Euler M The changing multiple sclerosis treatment landscape: impact of new drugs and treatment recommendations. Eur J Clin Pharmacol (2018) 74:663–70. 10.1007/s00228-018-2429-1 PMC5893684 29429031

[25] Straus Farber R Harel A Lublin F Novel Agents for Relapsing Forms of Multiple Sclerosis. Annu Rev Med (2016) 67:309–21. 10.1146/annurev-med-052814-023415 26394285

[26] Kim W Kim HJ Monoclonal Antibody Therapies for Multiple Sclerosis and Neuromyelitis Optica Spectrum Disorder. J Clin Neurol (2020) 16:355–68. 10.3988/jcn.2020.16.3.355 PMC7354979 32657055

[27] Myhr KM Torkildsen Ø Lossius A Bø L Holmøy T B cell depletion in the treatment of multiple sclerosis. Expert Opin Biol Ther (2019) 19:261–71. 10.1080/14712598.2019.1568407 30632834

[28] Ancau M Berthele A Hemmer B CD20 monoclonal antibodies for the treatment of multiple sclerosis: up-to-date. Expert Opin Biol Ther (2019) 19:829–43. 10.1080/14712598.2019.1611778 31027436

[29] Stys PK Tsutsui S Recent advances in understanding multiple sclerosis. F1000Res (2019) 8:F1000 Faculty Rev-2100. 10.12688/f1000research.20906.1

[30] Olsson T Barcellos LF Alfredsson L Interactions between genetic, lifestyle and environmental risk factors for multiple sclerosis. Nat Rev Neurol (2017) 13:25–36. 10.1038/nrneurol.2016.187 27934854

[31] Tarlinton RE Martynova E Rizvanov AA Khaiboullina S Verma S Role of Viruses in the Pathogenesis of Multiple Sclerosis. Viruses (2020) 12:E643. 10.3390/v12060643 32545816 PMC7354629

[32] Donati D Viral infections and multiple sclerosis. Drug Discov Today Dis Models (2020). 10.1016/j.ddmod.2020.02.003 PMC7102669 32292487

[33] Chan VS Epigenetics in Multiple Sclerosis. Adv Exp Med Biol (2020) 1253:309–74. 10.1007/978-981-15-3449-2_12 32445101

[34] De Silvestri A Capittini C Mallucci G Bergamaschi R Rebuffi C Pasi A The Involvement of HLA Class II Alleles in Multiple Sclerosis: A Systematic Review with Meta-analysis. Dis Markers (2019) 2019:1409069. 10.1155/2019/1409069 31781296 PMC6875418

[35] Moutsianas L Jostins L Beecham AH Dilthey AT Xifara DK Ban M Class II HLA interactions modulate genetic risk for multiple sclerosis. Nat Genet (2015) 47:1107–13. 10.1038/ng.3395 PMC4874245 26343388

[36] Lysandropoulos AP Mavroudakis N Pandolfo M El Hafsi K van Hecke W Maertens A HLA genotype as a marker of multiple sclerosis prognosis: A pilot study. J Neurol Sci (2017) 375:348–54. 10.1016/j.jns.2017.02.019 28320165

[37] Goodin DS Khankhanian P Gourraud PA Vince N Highly conserved extended haplotypes of the major histocompatibility complex and their relationship to multiple sclerosis susceptibility. PloS One (2018) 13:e0190043. 10.1371/journal.pone.0190043 29438392 PMC5810982

[38] Parnell GP Booth DR The Multiple Sclerosis (MS) Genetic Risk Factors Indicate both Acquired and Innate Immune Cell Subsets Contribute to MS Pathogenesis and Identify Novel Therapeutic Opportunities. Front Immunol (2017) 8:425:425. 10.3389/fimmu.2017.00425 28458668 PMC5394466

[39] Fogdell-Hahn A Ligers A Grønning M Hillert J Olerup O Multiple sclerosis: a modifying influence of HLA class I genes in an HLA class II associated autoimmune disease. Tissue Antigens (2000) 55:140–8. 10.1034/j.1399-0039.2000.550205.x 10746785

[40] International Multiple Sclerosis Genetics Consortium Wellcome Trust Case Control Consortium 2 Sawcer S Hellenthal G Pirinen M Spencer CC Genetic risk and a primary role for cell-mediated immune mechanisms in multiple sclerosis. Nature (2011) 476:214–9. 10.1038/nature10251 PMC3182531 21833088

[41] Pierrot-Deseilligny C Souberbielle JC Vitamin D and multiple sclerosis: An update. Mult Scler Relat Disord (2017) 14:35–45. 10.1016/j.msard.2017.03.014 28619429

[42] Miclea A Bagnoud M Chan A Hoepner R A Brief Review of the Effects of Vitamin D on Multiple Sclerosis. Front Immunol (2020) 11:781. 10.3389/fimmu.2020.00781 32435244 PMC7218089

[43] Rodney C Rodney S Millis RM Vitamin D and Demyelinating Diseases: Neuromyelitis Optica (NMO) and Multiple Sclerosis (MS). Autoimmune Dis (2020) 2020:8718736. 10.1155/2020/8718736 32373353 PMC7187724

[44] Hedström AK Olsson T Alfredsson L Smoking is a major preventable risk factor for multiple sclerosis. Mult Scler (2016) 22:1021–6. 10.1177/1352458515609794 26459151

[45] Arneth B Multiple Sclerosis and Smoking. Am J Med (2020) 133:783–8. 10.1016/j.amjmed.2020.03.008 32259516

[46] Degelman ML Herman KM Smoking and multiple sclerosis: A systematic review and meta-analysis using the Bradford Hill criteria for causation. Mult Scler Relat Disord (2017) 17:207–16. 10.1016/j.msard.2017.07.020 29055459

[47] Duscha A Gisevius B Hirschberg S Yissachar N Stangl GI Eilers E Propionic Acid Shapes the Multiple Sclerosis Disease Course by an Immunomodulatory Mechanism. Cell (2020) 180:1067–80.e16. 10.1016/j.cell.2020.02.035 32160527

[48] Brown J Quattrochi B Everett C Hong BY Cervantes J Gut commensals, dysbiosis, and immune response imbalance in the pathogenesis of multiple sclerosis. Mult Scler (2020) 1352458520928301. 10.1177/1352458520928301 32507072

[49] Mirza A Forbes JD Zhu F Bernstein CN Van Domselaar G Graham M The multiple sclerosis gut microbiota: A systematic review. Mult Scler Relat Disord (2020) 37:101427. 10.1016/j.msard.2019.101427 32172998

[50] Hedström AK Olsson T Alfredsson L Body mass index during adolescence, rather than childhood, is critical in determining MS risk. Mult Scler (2016) 22:878–83. 10.1177/1352458515603798 26362895

[51] Rasul T Frederiksen JL Link between overweight/obese in children and youngsters and occurrence of multiple sclerosis. J Neurol (2018) 265:2755–63. 10.1007/s00415-018-8869-9 29700643

[52] Novo AM Batista S Multiple Sclerosis: Implications of Obesity in Neuroinflammation. Adv Neurobiol (2017) 19:191–210. 10.1007/978-3-319-63260-5_8 28933066

[53] Hayes CE Ntambi JM Multiple Sclerosis: Lipids, Lymphocytes, and Vitamin D. Immunometabolism (2020) 2:e200019. 10.20900/immunometab20200019 32528735 PMC7289029

[54] Bar-Or A Pender MP Khanna R Steinman L Hartung HP Maniar T Epstein-Barr Virus in Multiple Sclerosis: Theory and Emerging Immunotherapies. Trends Mol Med (2020) 26:296–310. 10.1016/j.molmed.2019.11.003 31862243 PMC7106557

[55] Guan Y Jakimovski D Ramanathan M Weinstock-Guttman B Zivadinov R The role of Epstein-Barr virus in multiple sclerosis: from molecular pathophysiology to *in vivo* imaging. Neural Regener Res (2019) 14:373–86. 10.4103/1673-5374.245462 PMC6334604 30539801

[56] Ahmed SI Aziz K Gul A Samar SS Bareeqa SB Risk of Multiple Sclerosis in Epstein-Barr Virus Infection. Cureus (2019) 11:e5699. 10.7759/cureus.5699 31720167 PMC6823003

[57] Jouanguy E Béziat V Mogensen TH Casanova JL Tangye SG Zhang SY Human inborn errors of immunity to herpes viruses. Curr Opin Immunol (2020) 62:106–22. 10.1016/j.coi.2020.01.004 PMC7083196 32014647

[58] Siakallis G Spandidos DA Sourvinos G Herpesviridae and novel inhibitors. Antivir Ther (2009) 14:1051–64. 10.3851/IMP1467 20032535

[59] Arvin A Campadelli-Fiume G Mocarski E Moore PS Roizman B Whitley R Yamanishi K eds. Human Herpesviruses. Cambridge, UK: Cambridge University Press (2007). 21348071

[60] Majerciak V Yang W Zheng J Zhu J Zheng ZM A Genome-Wide Epstein-Barr Virus Polyadenylation Map and Its Antisense RNA to EBNA. J Virol (2019) 93(2):e01593–18. 10.1128/JVI.01593-18 PMC6321932 30355690

[61] Sakamoto K Sekizuka T Uehara T Hishima T Mine S Fukumoto H Next-generation sequencing of miRNAs in clinical samples of Epstein-Barr virus-associated B-cell lymphomas. Cancer Med (2017) 6:605–18. 10.1002/cam4.1006 PMC5345668 28181423

[62] Moss WN Lee N Pimienta G Steitz JA RNA families in Epstein-Barr virus. RNA Biol (2014) 11:10–7. 10.4161/rna.27488 PMC3929418 24441309

[63] Tarbouriech N Buisson M Géoui T Daenke S Cusack S Burmeister WP Structural genomics of the Epstein-Barr virus. Acta Crystallogr D Biol Crystallogr (2006) 62:1276–85. 10.1107/S0907444906030034 17001105

[64] Longnecker R Neipel F Introduction to the human γ-herpesviruses. In: Arvin, editor. Human Herpesviruses. Cambridge, UK: Cambridge University Press (2007). p. 341–59. 21348088

[65] Liu F Zhou ZH Comparative virion structures of human herpesviruses. In: Arvin, editor. Human Herpesviruses. Cambridge, UK: Cambridge University Press (2007). p. 27–43. 21348089

[66] Hammerschmidt W The Epigenetic Life Cycle of Epstein-Barr Virus. Curr Top Microbiol Immunol (2015) 390:103–17. 10.1007/978-3-319-22822-8_6 26424645

[67] Hatton OL Harris-Arnold A Schaffert S Krams SM Martinez OM The interplay between Epstein-Barr virus and B lymphocytes: implications for infection, immunity, and disease. Immunol Res (2014) 58:268–76. 10.1007/s12026-014-8496-1 PMC4199828 24619311

[68] Odumade OA Hogquist KA Balfour HH Progress and Problems in Understanding and Managing Primary Epstein-Barr Virus Infections. Clin Microbiol Rev (2011) 24:193–209. 10.1128/CMR.00044-10 21233512 PMC3021204

[69] Crawford DH Biology and disease associations of Epstein-Barr virus. Philos Trans R Soc Lond B Biol Sci (2001) 356:461–73. 10.1098/rstb.2000.0783 PMC1088438 11313005

[70] Thorley-Lawson DA Babcock GJ A model for persistent infection with Epstein-Barr virus: the stealth virus of human B cells. Life Sci (1999) 65:1433–53. 10.1016/s0024-3205(99)00214-3 10530796

[71] McKenzie J El-Guindy A Epstein-Barr Virus Lytic Cycle Reactivation. Curr Top Microbiol Immunol (2015) 391:237–61. 10.1007/978-3-319-22834-1_8 26428377

[72] Chakravorty A An Epigenetic Journey: Epstein-Barr Virus Transcribes Chromatinized and Subsequently Unchromatinized Templates during Its Lytic Cycle. Sugden B, Johannsen EC. J Virol (2019) 93:e02247–18. 10.1128/JVI.02247-18 PMC6450099 30700606

[73] Chiu YF Sugden B Epstein-Barr Virus: The Path from Latent to Productive Infection. Annu Rev Virol (2016) 3:359–72. 10.1146/annurev-virology-110615-042358 27578440

[74] Price AM Luftig MA Dynamic Epstein-Barr virus gene expression on the path to B-cell transformation. Adv Virus Res (2014) 88:279–313. 10.1016/B978-0-12-800098-4.00006-4 24373315 PMC4911173

[75] Münz C Latency and lytic replication in Epstein-Barr virus-associated oncogenesis. Nat Rev Microbiol (2019) 17:691–700. 10.1038/s41579-019-0249-7 31477887

[76] Kempkes B Robertson ES Epstein-Barr virus latency: current and future perspectives. Curr Opin Virol (2015) 14:138–44. 10.1016/j.coviro.2015.09.007 PMC5868753 26453799

[77] Murata T Regulation of Epstein-Barr virus reactivation from latency. Microbiol Immunol (2014) 58:307–17. 10.1111/1348-0421.12155 24786491

[78] Kerr JR Epstein-Barr virus (EBV) reactivation and therapeutic inhibitors. J Clin Pathol (2019) 72:651–8. 10.1136/jclinpath-2019-205822 31315893

[79] Kenney SC Reactivation and lytic replication of EBV. In: Arvin, editor. Human Herpesviruses. Cambridge, UK: Cambridge University Press (2007). p. 403–33. 21348125

[80] Liebermann PM Hu J Renne R Maintenance and replication during latency. In: Arvin, editor. Human Herpesviruses. Cambridge, UK: Cambridge University Press (2007). p. 379–402. 21348090

[81] Ressing ME van Gent M Gram AM Hooykaas MJ Piersma SJ Wiertz EJ Immune Evasion by Epstein-Barr Virus. Curr Top Microbiol Immunol (2015) 391:355–81. 10.1007/978-3-319-22834-1_12 26428381

[82] Wang M Yu F Wu W Wang Y Ding H Qian L Epstein-Barr virus-encoded microRNAs as regulators in host immune responses. Int J Biol Sci (2018) 14:565–76. 10.7150/ijbs.24562 PMC5968849 29805308

[83] Iizasa H Kim H Kartika AV Kanehiro Y Yoshiyama H Role of Viral and Host microRNAs in Immune Regulation of Epstein-Barr Virus-Associated Diseases. Front Immunol (2020) 11:367. 10.3389/fimmu.2020.00367 32194570 PMC7062708

[84] Albanese M Tagawa T Buschle A Hammerschmidt W MicroRNAs of Epstein- Barr Virus Control Innate and Adaptive Antiviral Immunity. J Virol (2017) 91:e01667–16. 10.1128/JVI.01667-16 PMC5533892 28592533

[85] Rowe M Zuo J Immune responses to Epstein-Barr virus: molecular interactions in the virus evasion of CD8+ T cell immunity. Microbes Infect (2010) 12:173–81. 10.1016/j.micinf.2009.12.001 PMC2832755 20004735

[86] Means RE Lang SM Jung JU Human Herpesviruses. In: Arvin A Campadelli-Fiume G Mocarski E Moore PS Roizman B Whitley R editors. Cambridge, UK: Cambridge University Press (2007). pp 559–86.

[87] Middeldorp JM Epstein-Barr Virus-Specific Humoral Immune Responses in Health and Disease. Curr Top Microbiol Immunol (2015) 391:289–323. 10.1007/978-3-319-22834-1_10 26428379

[88] Latour S Fischer A Signaling pathways involved in the T-cell-mediated immunity against Epstein-Barr virus: Lessons from genetic diseases. Immunol Rev (2019) 291:174–89. 10.1111/imr.12791 31402499

[89] Chijioke O Azzi T Nadal D Münz C Innate immune responses against Epstein Barr virus infection. J Leukoc Biol (2013) 94:1185–90. 10.1189/jlb.0313173 PMC3828602 23812328

[90] Münz C Epstein-Barr Virus-Specific Immune Control by Innate Lymphocytes. Front Immunol (2017) 8:1658. 10.3389/fimmu.2017.01658 29225606 PMC5705607

[91] Jangra S Yuen KS Botelho MG Jin DY Epstein-Barr Virus and Innate Immunity: Friends or Foes? Microorganisms (2019) 7:183. 10.3390/microorganisms7060183 PMC6617214 31238570

[92] Fares S Spiess K Olesen ETB Zuo J Jackson S Kledal TN Distinct Roles of Extracellular Domains in the Epstein-Barr Virus-Encoded BILF1 Receptor for Signaling and Major Histocompatibility Complex Class I Downregulation. mBio (2019) 10:e01707–18. 10.1128/mBio.01707-18 PMC6336419 30647152

[93] Albanese M Tagawa T Bouvet M Maliqi L Lutter D Hoser J Epstein-Barr virus microRNAs reduce immune surveillance by virus-specific CD8+ T cells. Proc Natl Acad Sci USA (2016) 113:E6467–75. 10.1073/pnas.1605884113 PMC5081573 27698133

[94] Quinn LL Williams LR White C Forrest C Zuo J Rowe M The Missing Link in Epstein-Barr Virus Immune Evasion: the BDLF3 Gene Induces Ubiquitination and Downregulation of Major Histocompatibility Complex Class I (MHC-I) and MHC-II. J Virol (2015) 90:356–67. 10.1128/JVI.02183-15 PMC4702578 26468525

[95] Lin JH Lin JY Chou YC Chen MR Yeh TH Lin CW Epstein-Barr virus LMP2A suppresses MHC class II expression by regulating the B-cell transcription factors E47 and PU.1. Blood (2015) 125:2228–38. 10.1182/blood-2014-08-594689 25631773

[96] Croft NP Shannon-Lowe C Bell AI Horst D Kremmer E Ressing ME Stage-specific inhibition of MHC class I presentation by the Epstein-Barr virus BNLF2a protein during virus lytic cycle. PloS Pathog (2009) 5:e1000490. 10.1371/journal.ppat.1000490 19557156 PMC2695766

[97] Li D Qian L Chen C Shi M Yu M Hu M Down-regulation of MHC class II expression through inhibition of CIITA transcription by lytic transactivator Zta during Epstein-Barr virus reactivation. J Immunol (2009) 182:1799–809. 10.4049/jimmunol.0802686 19201831

[98] Zuo J Currin A Griffin BD Shannon-Lowe C Thomas WA Ressing ME The Epstein-Barr virus G-protein-coupled receptor contributes to immune evasion by targeting MHC class I molecules for degradation. PloS Pathog (2009) 5:e1000255. 10.1371/journal.ppat.1000255 19119421 PMC2603334

[99] Ressing ME Horst D Griffin BD Tellam J Zuo J Khanna R Epstein-Barr virus evasion of CD8(+) and CD4(+) T cell immunity via concerted actions of multiple gene products. Semin Cancer Biol (2008) 18:397–408. 10.1016/j.semcancer.2008.10.008 18977445

[100] Guerreiro-Cacais AO Uzunel M Levitskaya J Levitsky V Inhibition of heavy chain and beta2-microglobulin synthesis as a mechanism of major histocompatibility complex class I downregulation during Epstein-Barr virus replication. J Virol (2007) 81:1390–400. 10.1128/JVI.01999-06 PMC1797541 17108039

[101] Pappworth IY Wang EC Rowe M The switch from latent to productive infection in epstein-barr virus-infected B cells is associated with sensitization to NK cell killing. J Virol (2007) 81:474–82. 10.1128/JVI.01777-06 PMC1797427 17079298

[102] Keating S Prince S Jones M Rowe M The lytic cycle of Epstein-Barr virus is associated with decreased expression of cell surface major histocompatibility complex class I and class II molecules. J Virol (2002) 76:8179–88. 10.1128/jvi.76.16.8179-8188.2002 PMC155144 12134023

[103] Dunmire SK Hogquist KA Balfour HH Infectious Mononucleosis. Curr Top Microbiol Immunol (2015) 390:211–40. 10.1007/978-3-319-22822-8_9 PMC4670567 26424648

[104] Dunmire SK Verghese PS Balfour HH Jr Primary Epstein-Barr virus infection. J Clin Virol (2018) 102:84–92. 10.1016/j.jcv.2018.03.001 29525635

[105] Farrell PJ Epstein-Barr Virus and Cancer. Annu Rev Pathol (2019) 14:29–53. 10.1146/annurev-pathmechdis-012418-013023 30125149

[106] Rostgaard K Balfour HH Jr Jarrett R Erikstrup C Pedersen O Ullum H Primary Epstein-Barr virus infection with and without infectious mononucleosis. PloS One (2019) 14:e0226436. 10.1371/journal.pone.0226436 31846480 PMC6917282

[107] Hue SS Oon ML Wang S Tan SY Ng SB Epstein-Barr virus-associated T- and NK-cell lymphoproliferative diseases: an update and diagnostic approach. Pathology (2020) 52:111–27. 10.1016/j.pathol.2019.09.011 31767131

[108] Iwatsuki K Miyake T Hirai Y Yamamoto T Hydroa vacciniforme: a distinctive form of Epstein-Barr virus-associated T-cell lymphoproliferative disorders. Eur J Dermatol (2019) 29:21–8. 10.1684/ejd.2018.3490 30998212

[109] Jha HC Pei Y Robertson ES Epstein-Barr Virus: Diseases Linked to Infection and Transformation. Front Microbiol (2016) 7:1602. 10.3389/fmicb.2016.01602 27826287 PMC5078142

[110] Saha A Robertson ES Epstein-Barr virus-associated B-cell lymphomas: pathogenesis and clinical outcomes. Clin Cancer Res (2011) 17:3056–63. 10.1158/1078-0432.CCR-10-2578 PMC4287361 21372216

[111] Kutok JL Wang F Spectrum of Epstein-Barr virus-associated diseases. Annu Rev Pathol (2006) 1:375–404. 10.1146/annurev.pathol.1.110304.100209 18039120

[112] Draborg AH Duus K Houen G Epstein-Barr virus in systemic autoimmune diseases. Clin Dev Immunol (2013) 2013:535738. 10.1155/2013/535738 24062777 PMC3766599

[113] Hohlfeld R Dornmair K Meinl E Wekerle H The search for the target antigens of multiple sclerosis, part 1: autoreactive CD4+ T lymphocytes as pathogenic effectors and therapeutic targets. Lancet Neurol (2016) 15:198–209. 10.1016/S1474-4422(15)00334-8 26724103

[114] Pröbstel AK Sanderson NS Derfuss T B Cells and Autoantibodies in Multiple Sclerosis. Int J Mol Sci (2015) 16:16576–92. 10.3390/ijms160716576 PMC4519967 26197319

[115] Weber MS Hemmer B Cepok S The role of antibodies in multiple sclerosis. Biochim Biophys Acta (2011) 1812:239–45. 10.1016/j.bbadis.2010.06.009 20600871

[116] Reindl M Linington C Brehm U Egg R Dilitz E Deisenhammer F Antibodies against the myelin oligodendrocyte glycoprotein and the myelin basic protein in multiple sclerosis and other neurological diseases: a comparative study. Brain (1999) 122:2047–56. 10.1093/brain/122.11.2047 10545390

[117] Derfuss T Kuhle J Lindberg R Kappos L Natalizumab therapy for multiple sclerosis. Semin Neurol (2013) 33:26–36. 10.1055/s-0033-1343793 23709210

[118] Shannon-Lowe C Rowe M Epstein Barr virus entry; kissing and conjugation. Curr Opin Virol (2014) 4:78–84. 10.1016/j.coviro.2013.12.001 24553068

[119] Kishishita M Yanase S Ito Y Activation of Epstein-Barr virus expression in human lymphoblastoid P3HR-1 and Raji cells with propionic acid and with culture fluids of propionic acid-producing anaerobes. Cancer Lett (1982) 16:117–20. 10.1016/0304-3835(82)90051-9 6290028

[120] Pender MP CD8+ T-Cell Deficiency, Epstein-Barr Virus Infection, Vitamin D Deficiency, and Steps to Autoimmunity: A Unifying Hypothesis. Autoimmune Dis (2012) 2012:189096. 10.1155/2012/189096 22312480 PMC3270541

[121] Jacobs BM Giovannoni G Cuzick J Dobson R Systematic review and meta-analysis of the association between Epstein-Barr virus, multiple sclerosis and other risk factors. Mult Scler (2020) 26:1281–97. 10.1177/1352458520907901. 1352458520907901. PMC7543008 32202208

[122] Hedström AK Huang J Michel A Butt J Brenner N Hillert J High Levels of Epstein-Barr Virus Nuclear Antigen-1-Specific Antibodies and Infectious Mononucleosis Act Both Independently and Synergistically to Increase Multiple Sclerosis Risk. Front Neurol (2020) 10:1368. 10.3389/fneur.2019.01368 32038456 PMC6992610

[123] Tselis A Epstein-Barr virus cause of multiple sclerosis. Curr Opin Rheumatol (2012) 24:424–8. 10.1097/BOR.0b013e3283542cf8 22617821

[124] Ruprecht K Wildemann B Jarius S Low intrathecal antibody production despite high seroprevalence of Epstein-Barr virus in multiple sclerosis: a review of the literature. J Neurol (2018) 265:239–52. 10.1007/s00415-017-8656-z 29098417

[125] Kuri A Jacobs BM Vickaryous N Pakpoor J Middeldorp J Giovannoni G Epidemiology of Epstein-Barr virus infection and infectious mononucleosis in the United Kingdom. BMC Public Health (2020) 20:912. 10.1186/s12889-020-09049-x 32532296 PMC7291753

[126] Choi A Marcus K Pohl D Eyck PT Balfour H Jr Jackson JB Epstein-Barr virus infection status among first year undergraduate university students. J Am Coll Health (2020) 1–4. 10.1080/07448481.2020.1726927 PMC7832088 32101103

[127] Winter JR Jackson C Lewis JE Taylor GS Thomas OG Stagg HR Predictors of Epstein-Barr virus serostatus and implications for vaccine policy: A systematic review of the literature. J Glob Health (2020) 10:10404. 10.7189/jogh.10.010404 PMC7125428 32257152

[128] Fisher KS Cuascut FX Rivera VM Hutton GJ Current Advances in Pediatric Onset Multiple Sclerosis. Biomedicines (2020) 8:71. 10.3390/biomedicines8040071 PMC7235875 32231060

[129] Banwell B Krupp L Kennedy J Tellier R Tenembaum S Ness J Clinical features and viral serologies in children with multiple sclerosis: a multinational observational study. Lancet Neurol (2007) 6:773–81. 10.1016/S1474-4422(07)70196-5 17689148

[130] Pakpoor J Disanto G Gerber JE Dobson R Meier UC Giovannoni G The risk of developing multiple sclerosis in individuals seronegative for Epstein-Barr virus: a meta-analysis. Mult Scler (2013) 19:162–6. 10.1177/1352458512449682 22740437

[131] Lünemann JD Huppke P Roberts S Brück W Gärtner J Münz C Broadened and elevated humoral immune response to EBNA1 in pediatric multiple sclerosis. Neurology (2008) 71:1033–5. 10.1212/01.wnl.0000326576.91097.87 PMC2676958 18809840

[132] Pohl D Krone B Rostasy K Kahler E Brunner E Lehnert M High seroprevalence of Epstein-Barr virus in children with multiple sclerosis. Neurology (2006) 67:2063–5. 10.1212/01.wnl.0000247665.94088.8d 17159123

[133] Abrahamyan S Eberspächer B Hoshi MM Aly L Luessi F Groppa S German Competence Network Multiple Sclerosis (KKNMS); Other members of the KKNMS that acted as collaborators in this study. Complete Epstein-Barr virus seropositivity in a large cohort of patients with early multiple sclerosis. J Neurol Neurosurg Psychiatry (2020) 91:681–6. 10.1136/jnnp-2020-322941 PMC7361012 32371533

[134] Almohmeed YH Avenell A Aucott L Vickers MA Systematic review and meta-analysis of the sero-epidemiological association between Epstein Barr virus and multiple sclerosis. PloS One (2013) 8:e61110. 10.1371/journal.pone.0061110 23585874 PMC3621759

[135] Langer-Gould A Wu J Lucas R Smith J Gonzales E Amezcua L Epstein-Barr virus, cytomegalovirus, and multiple sclerosis susceptibility: A multiethnic study. Neurology (2017) 89:1330–7. 10.1212/WNL.0000000000004412 PMC5649756 28855411

[136] Sisay S Lopez-Lozano L Mickunas M Quiroga-Fernández A Palace J Warnes G Untreated relapsing remitting multiple sclerosis patients show antibody production against latent Epstein Barr Virus (EBV) antigens mainly in the periphery and innate immune IL-8 responses preferentially in the CNS. J Neuroimmunol (2017) 306:40–5. 10.1016/j.jneuroim.2017.02.017 28385186

[137] Dooley MM de Gannes SL Fu KA Lindsey JW The increased antibody response to Epstein-Barr virus in multiple sclerosis is restricted to selected virus proteins. J Neuroimmunol (2016) 299:147–51. 10.1016/j.jneuroim.2016.08.016 27725113

[138] Hedström AK Lima Bomfim I Hillert J Olsson T Alfredsson L Obesity interacts with infectious mononucleosis in risk of multiple sclerosis. Eur J Neurol (2015) 22:578–e38. 10.1111/ene.12620 25530445 PMC4365756

[139] Hedström AK Huang J Brenner N Butt J Hillert J Waterboer T Smoking and Epstein-Barr virus infection in multiple sclerosis development. Sci Rep (2020) 10:10960. 10.1038/s41598-020-67883-w 32620875 PMC7335184

[140] Holden DW Gold J Hawkes CH Giovannoni G Saxton JM Carter A Epstein Barr virus shedding in multiple sclerosis: Similar frequencies of EBV in saliva across separate patient cohorts. Mult Scler Relat Disord (2018) 25:197–9. 10.1016/j.msard.2018.07.041 30099206

[141] Hon GM Hassan MS van Rensburg SJ Erasmus RT Matsha TE Assessment of Epstein-Barr virus in blood from patients with multiple sclerosis. Metab Brain Dis (2012) 27:311–8. 10.1007/s11011-012-9292-z 22407028

[142] Lindsey JW Hatfield LM Crawford MP Patel S Quantitative PCR for Epstein-Barr virus DNA and RNA in multiple sclerosis. Mult Scler (2009) 15:153–8. 10.1177/1352458508097920 18845656

[143] Mechelli R Manzari C Policano C Annese A Picardi E Umeton R Epstein-Barr virus genetic variants are associated with multiple sclerosis. Neurology (2015) 84:1362–8. 10.1212/WNL.0000000000001420 PMC4388746 25740864

[144] Santón A Cristóbal E Aparicio M Royuela A Villar LM Alvarez-Cermeño JC High frequency of co-infection by Epstein-Barr virus types 1 and 2 in patients with multiple sclerosis. Mult Scler (2011) 17:1295–300. 10.1177/1352458511411063 21757537

[145] Veroni C Serafini B Rosicarelli B Fagnani C Aloisi F Transcriptional profile and Epstein-Barr virus infection status of laser-cut immune infiltrates from the brain of patients with progressive multiple sclerosis. J Neuroinflammation (2018) 15:18. 10.1186/s12974-017-1049-5 29338732 PMC5771146

[146] Hassani A Corboy JR Al-Salam S Khan G Epstein-Barr virus is present in the brain of most cases of multiple sclerosis and may engage more than just B cells. PloS One (2018) 13:e0192109. 10.1371/journal.pone.0192109 29394264 PMC5796799

[147] Willis SN Stadelmann C Rodig SJ Caron T Gattenloehner S Mallozzi SS Epstein-Barr virus infection is not a characteristic feature of multiple sclerosis brain. Brain (2009) 132:3318–28. 10.1093/brain/awp200 PMC2792367 19638446

[148] Sargsyan SA Shearer AJ Ritchie AM Burgoon MP Anderson S Hemmer B Absence of Epstein-Barr virus in the brain and CSF of patients with multiple sclerosis. Neurology (2010) 74:1127–35. 10.1212/WNL.0b013e3181d865a1 PMC2865779 20220124

[149] Magliozzi R Serafini B Rosicarelli B Chiappetta G Veroni C Reynolds R Aloisi F B-cell enrichment and Epstein-Barr virus infection in inflammatory cortical lesions in secondary progressive multiple sclerosis. J Neuropathol Exp Neurol (2013) 72:29–41. 10.1097/NEN.0b013e31827bfc62 23242282

[150] Tarlinton RE Martynova E Rizvanov AA Khaiboullina S Verma S Role of Viruses in the Pathogenesis of Multiple Sclerosis. Viruses (2020) 12:E643. 10.3390/v12060643 32545816 PMC7354629

[151] Geginat J Paroni M Pagani M Galimberti D De Francesco R Scarpini E The Enigmatic Role of Viruses in Multiple Sclerosis: Molecular Mimicry or Disturbed Immune Surveillance? Trends Immunol (2017) 38:498–512. 10.1016/j.it.2017.04.006 28549714 PMC7185415

[152] Mentis AA Dardiotis E Grigoriadis N Petinaki E Hadjigeorgiou GM Viruses and Multiple Sclerosis: From Mechanisms and Pathways to Translational Research Opportunities. Mol Neurobiol (2017) 54:3911–23. 10.1007/s12035-017-0530-6 28455696

[153] Virtanen JO Jacobson S Viruses and multiple sclerosis. CNS Neurol Disord Drug Targets (2012) 11:528–44. 10.2174/187152712801661220 PMC4758194 22583435

[154] Owens GP Gilden D Burgoon MP Yu X Bennett JL Viruses and multiple sclerosis. Neuroscientist (2011) 17:659–76. 10.1177/1073858411386615 PMC3293404 22130640

[155] Pender MP Csurhes PA Burrows JM Burrows SR Defective T-cell control of Epstein-Barr virus infection in multiple sclerosis. Clin Transl Immunol (2017) 6:e126. 10.1038/cti.2016.87 PMC5292561 28197337

[156] Serafini B Rosicarelli B Veroni C Mazzola GA Aloisi F Epstein-Barr Virus-Specific CD8 T Cells Selectively Infiltrate the Brain in Multiple Sclerosis and Interact Locally with Virus-Infected Cells: Clue for a Virus-Driven Immunopathological Mechanism. J Virol (2019) 93:e00980–19. 10.1128/JVI.00980-19 PMC6880158 31578295

[157] van Nierop GP Mautner J Mitterreiter JG Hintzen RQ Verjans GM Intrathecal CD8 T-cells of multiple sclerosis patients recognize lytic Epstein-Barr virus proteins. Mult Scler (2016) 22:279–91. 10.1177/1352458515588581 26041797

[158] Cencioni MT Magliozzi R Nicholas R Ali R Malik O Reynolds R Programmed death 1 is highly expressed on CD8(+) CD57(+) T cells in patients with stable multiple sclerosis and inhibits their cytotoxic response to Epstein-Barr virus. Immunology (2017) 152:660–76. 10.1111/imm.12808 PMC5680058 28767147

[159] Sambucci M Gargano F De Rosa V De Bardi M Picozza M Placido R FoxP3 isoforms and PD-1 expression by T regulatory cells in multiple sclerosis. Sci Rep (2018) 8:3674. 10.1038/s41598-018-21861-5 29487369 PMC5829149

[160] Wanleenuwat P Iwanowski P Role of B cells and antibodies in multiple sclerosis. Mult Scler Relat Disord (2019) 36:101416. 10.1016/j.msard.2019.101416 31577986

[161] Levin MC Lee S Gardner LA Shin Y Douglas JN Cooper C Autoantibodies to Non-myelin Antigens as Contributors to the Pathogenesis of Multiple Sclerosis. J Clin Cell Immunol (2013) 4. 10.4172/2155-9899.1000148 PMC3866957 24363960

[162] Vyshkina T Kalman B Autoantibodies and neurodegeneration in multiple sclerosis. Lab Invest (2008) 88:796–807. 10.1038/labinvest.2008.53 18521063

[163] Waegemans T Auto-antibodies in multiple sclerosis: an hypothesis. BioMed Pharmacother (2004) 58:282–5. 10.1016/j.biopha.2004.04.003 15194163

[164] Navas-Madroñal M Valero-Mut A Martínez-Zapata MJ Simón-Talero MJ Figueroa S Vidal-Fernández N Absence of antibodies against KIR4.1 in multiple sclerosis: A three-technique approach and systematic review. PloS One (2017) 12:e0175538. 10.1371/journal.pone.0175538 28414733 PMC5393569

[165] Funaro M Messina M Shabbir M Wright P Najjar S Tabansky I The role of B cells in multiple sclerosis: more than antibodies. Discovery Med (2016) 22:251–5. 28009967

[166] Merashli M Alves JD Gentile F Ames PRJ Relevance of antiphospholipid antibodies in multiple sclerosis: A systematic review and meta analysis. Semin Arthritis Rheum (2017) 46:810–8. 10.1016/j.semarthrit.2016.09.010 27908533

[167] Zhou D Srivastava R Nessler S Grummel V Sommer N Brück W Identification of a pathogenic antibody response to native myelin oligodendrocyte glycoprotein in multiple sclerosis. Proc Natl Acad Sci USA (2006) 103:19057–62. 10.1073/pnas.0607242103 PMC1748176 17142321

[168] Häusser-Kinzel S Weber MS The Role of B Cells and Antibodies in Multiple Sclerosis, Neuromyelitis Optica, and Related Disorders. Front Immunol (2019) 10:201. 10.3389/fimmu.2019.00201 30800132 PMC6375838

[169] Sospedra M Sospedra M B cells in multiple sclerosis. Curr Opin Neurol (2018) 31:256–62. 10.1097/WCO.000000000000563 29629941

[170] Racke MK The role of B cells in multiple sclerosis: rationale for B-cell-targeted therapies. Curr Opin Neurol (2008) 21 Suppl 1:S9–S18. 10.1097/01.wco.0000313359.61176.15 18388801

[171] Franciotta D Salvetti M Lolli F Serafini B Aloisi F B cells and multiple sclerosis. Lancet Neurol (2008) 7:852–8. 10.1016/S1474-4422(08)70192-3 18703007

[172] Ayoglu B Mitsios N Kockum I Khademi M Zandian A Sjöberg R Anoctamin 2 identified as an autoimmune target in multiple sclerosis. Proc Natl Acad Sci USA (2016) 113:2188–93. 10.1073/pnas.1518553113 PMC4776531 26862169

[173] Sabatino JJ Jr Zamvil SS T cells take aim at a ubiquitous autoantigen in multiple sclerosis. Sci Transl Med (2018) 10:eaau8826. 10.1126/scitranslmed.aau8826 30305455

[174] Li YF Zhang SX Ma XW Xue YL Gao C Li XY The proportion of peripheral regulatory T cells in patients with Multiple Sclerosis: A meta-analysis. Mult Scler Relat Disord (2019) 28:75–80. 10.1016/j.msard.2018.12.019 30572285

[175] Seidkhani-Nahal A Noori-Zadeh A Bakhtiyari S Khosravi A Frequency of CD8(+) regulatory T cells in the multiple sclerosis patients: a systematic review and meta-analysis. Acta Neurol Belg (2019) 119:61–8. 10.1007/s13760-018-1028-3 30324330

[176] Kaskow BJ Baecher-Allan C Effector T Cells in Multiple Sclerosis. Cold Spring Harb Perspect Med (2018) 8:a029025. 10.1101/cshperspect.a029025 29358315 PMC5880159

[177] Danikowski KM Jayaraman S Prabhakar BS Regulatory T cells in multiple sclerosis and myasthenia gravis. J Neuroinflammation (2017) 14:117. 10.1186/s12974-017-0892-8 28599652 PMC5466736

[178] Bianchini E De Biasi S Simone AM Ferraro D Sola P Cossarizza A Invariant natural killer T cells and mucosal-associated invariant T cells in multiple sclerosis. Immunol Lett (2017) 183:1–7. 10.1016/j.imlet.2017.01.009 28119072

[179] Hohlfeld R Dornmair K Meinl E Wekerle H The search for the target antigens of multiple sclerosis, part 2: CD8+ T cells, B cells, and antibodies in the focus of reverse-translational research. Lancet Neurol (2016) 15:317–31. 10.1016/S1474-4422(15)00313-0 26724102

[180] Dhaeze T Hellings N Circulating Follicular Regulatory T Cells in Autoimmune Diseases and Their Waning in Multiple Sclerosis. Crit Rev Immunol (2016) 36:511–22. 10.1615/CritRevImmunol.2017019850 28845757

[181] Sinha S Boyden AW Itani FR Crawford MP Karandikar NJ CD8(+) T-Cells as Immune Regulators of Multiple Sclerosis. Front Immunol (2015) 6:619. 10.3389/fimmu.2015.00619 26697014 PMC4674574

[182] Elong Ngono A Pettré S Salou M Bahbouhi B Soulillou JP Brouard S Frequency of circulating autoreactive T cells committed to myelin determinants in relapsing-remitting multiple sclerosis patients. Clin Immunol (2012) 144:117–26. 10.1016/j.clim.2012.05.009 22717772

[183] McCormack PL Natalizumab: a review of its use in the management of relapsing-remitting multiple sclerosis. Drugs (2013) 73:1463–81. 10.1007/s40265-013-0102-7 23912625

[184] Zivadinov R Ramanathan M Hagemeier J Bergsland N Ramasamy DP Durfee J Teriflunomide’s effect on humoral response to Epstein-Barr virus and development of cortical gray matter pathology in multiple sclerosis. Mult Scler Relat Disord (2019) 36:101388. 10.1016/j.msard.2019.101388 31525628

[185] Pender MP Csurhes PA Smith C Douglas NL Neller MA Matthews KK Epstein-Barr virus-specific T cell therapy for progressive multiple sclerosis. JCI Insight (2018) 3:e124714. 10.1172/jci.insight.124714 PMC6302936 30429369

[186] Pender MP Csurhes PA Smith C Beagley L Hooper KD Raj M Epstein-Barr virus-specific adoptive immunotherapy for progressive multiple sclerosis. Mult Scler (2014) 20:1541–4. 10.1177/1352458514521888 PMC4230458 24493474

[187] Pender MP Csurhes PA Pfluger CM Burrows SR Deficiency of CD8+ effector memory T cells is an early and persistent feature of multiple sclerosis. Mult Scler (2014) 20:1825–32. 10.1177/1352458514536252 PMC4361480 24842963

[188] Lucas RM Hughes AM Lay ML Ponsonby AL Dwyer DE Taylor BV Epstein-Barr virus and multiple sclerosis. J Neurol Neurosurg Psychiatry (2011) 82:1142–8. 10.1136/jnnp-2011-300174 21836034

[189] Pender MP Csurhes PA Pfluger CM Burrows SR Decreased CD8+ T cell response to Epstein-Barr virus infected B cells in multiple sclerosis is not due to decreased HLA class I expression on B cells or monocytes. BMC Neurol (2011) 11:95. 10.1186/1471-2377-11-95 21810280 PMC3163532

[190] Pender MP Csurhes PA Pfluger CM Burrows SR CD8 T cell deficiency impairs control of Epstein–Barr virus and worsens with age in multiple sclerosis. J Neurol Neurosurg Psychiatry (2012) 83:353–4. 10.1136/jnnp-2011-300213 PMC3277686 21791511

[191] Pender MP The essential role of Epstein-Barr virus in the pathogenesis of multiple sclerosis. Neuroscientist (2011) 17:351–67. 10.1177/1073858410381531 PMC3764840 21075971

[192] Brennan RM Burrows JM Bell MJ Bromham L Csurhes PA Lenarczyk A Strains of Epstein-Barr virus infecting multiple sclerosis patients. Mult Scler (2010) 16:643–51. 10.1177/1352458510364537 20350958

[193] Pender MP Does Epstein-Barr virus infection in the brain drive the development of multiple sclerosis? Brain (2009) 132:3196–8. 10.1093/brain/awp312 20008341

[194] Pender MP Preventing and curing multiple sclerosis by controlling Epstein-Barr virus infection. Autoimmun Rev (2009) 8:563–8. 10.1016/j.autrev.2009.01.017 19254880

[195] Pender MP Csurhes PA Lenarczyk A Pfluger CM Burrows SR Decreased T cell reactivity to Epstein-Barr virus infected lymphoblastoid cell lines in multiple sclerosis. J Neurol Neurosurg Psychiatry (2009) 80:498–505. 10.1136/jnnp.2008.161018 19015225 PMC2663364

[196] Greer JM Pender MP The presence of glutamic acid at positions 71 or 74 in pocket 4 of the HLA-DRbeta1 chain is associated with the clinical course of multiple sclerosis. J Neurol Neurosurg Psychiatry (2005) 76:656–62. 10.1136/jnnp.2004.042168 PMC1739634 15834022

[197] Pender MP Infection of autoreactive B lymphocytes with EBV, causing chronic autoimmune diseases. Trends Immunol (2003) 24:584–8. 10.1016/j.it.2003.09.005 14596882

[198] Otto C Hofmann J Ruprecht K Antibody producing B lineage cells invade the central nervous system predominantly at the time of and triggered by acute Epstein-Barr virus infection: A hypothesis on the origin of intrathecal immunoglobulin synthesis in multiple sclerosis. Med Hypotheses (2016) 91:109–13. 10.1016/j.mehy.2016.04.025 27142157

[199] Laurence M Benito-León J Epstein-Barr virus and multiple sclerosis: Updating Pender’s hypothesis. Mult Scler Relat Disord (2017) 16:8–14. 10.1016/j.msard.2017.05.009 28755684

[200] Kearns PKA Casey HA Leach JP Hypothesis: Multiple sclerosis is caused by three-hits, strictly in order, in genetically susceptible persons. Mult Scler Relat Disord (2018) 24:157–74. 10.1016/j.msard.2018.06.014 30015080

[201] van Langelaar J Rijvers L Smolders J van Luijn MM B and T Cells Driving Multiple Sclerosis: Identity, Mechanisms and Potential Triggers. Front Immunol (2020) 11:760. 10.3389/fimmu.2020.00760 32457742 PMC7225320

